# Passive immunisation of convalescent human anti-Zika plasma protects against challenge with New World Zika virus in cynomolgus macaques

**DOI:** 10.1038/s41541-020-00234-y

**Published:** 2020-09-15

**Authors:** Neil Berry, Sarah Kempster, Claire Ham, Adrian Jenkins, Jo Hall, Mark Page, Giada Mattiuzzo, Yemisi Adedeji, Roger Hewson, Elaine Giles, Debbie Ferguson, Neil Almond

**Affiliations:** 1 Division of Infectious Disease Diagnostics, National Institute for Biological Standards and Control, Blanche Lane, South Mimms, Herts EN6 3QG UK; 2 Division of Virology, National Institute for Biological Standards and Control, Blanche Lane, South Mimms, Herts EN6 3QG UK; 3 Virology and Pathogenesis, National Infection Service, PHE-Porton, Manor Farm Rd, Porton Down, Salisbury SP4 0JG UK

**Keywords:** Microbiology, Diseases

## Abstract

Zika virus (ZIKV) causes neurological complications in susceptible individuals, highlighted in the recent South American epidemic. Natural ZIKV infection elicits host responses capable of preventing subsequent re-infection, raising expectations for effective vaccination. Defining protective immune correlates will inform viral intervention strategies, particularly vaccine development. Non-human primate (NHP) species are susceptible to ZIKV and represent models for vaccine development. The protective efficacy of a human anti-ZIKV convalescent plasma pool (16/320-14) developed as a candidate reference material for a WHO International Standard was evaluated in macaques. Convalescent plasma administered to four cynomolgus macaques (*Macaca fascicularis*) intra-peritoneally 24 hrs prior to sub-cutaneous challenge with 10^3^ pfu ZIKV_PRVABC59_ protected against detectable infection, with absence of detectable ZIKV RNA in blood and lymphoid tissues. Passively immunised anti-ZIKV immunoglobulin administered prior to time of challenge remained present only at very low levels 42 days post-challenge. Absence of de novo antibody responses in passively immunised macaques indicate sterilising immunity compared with naïve challenge controls that exhibited active ZIKV-specific IgM and IgG responses post-challenge. Demonstration that the presence of convalescent anti-ZIKV at levels of 400 IU/mL neutralising antibody protects against virus challenge provides a scientific framework for development of anti-ZIKV vaccines and facilitates regulatory approval.

## Introduction

Zika virus (ZIKV) is a Flavivirus and newly re-emergent human pathogen now prevalent in parts of Central and South America^1^, having spread from Africa and Asia across the Pacific Ocean in the previous decade^2^. In 2015, ZIKV erupted as a major epidemic in South and Central America, subsequently identified as a global Public Health Emergency of International Concern by the World Health Organisation (WHO). In South America, ZIKV was linked to significant neurological complications, notably microcephaly, in children born to Zika positive mothers^3–6^ and a range of peripheral neuropathy conditions in adults including Guillain-Barre Syndrome^7,8^. International initiatives were established to better understand virus pathogenesis and develop strategies to combat future outbreaks. Studies conducted in different NHP species have elucidated pathogenesis whereby ZIKV is no longer regarded simply as a self-limiting acute infection but persisting viral signals have been detected in multiple anatomical sites many weeks after the initial infection has cleared from the blood^9–16^.

ZIKV has a broad host range capable of productively infecting a spectrum of higher mammals including humans and both Old and New World monkey species^16,17^. Antibody responses to major viral proteins (e.g., NS-1 and envelope antigens), particularly a broadly neutralising antibody response, are associated with recovery from ZIKV infection that appears to confer protective immunity against re-infection, with a lack of new infections in exposed, immune populations as confirmed by epidemiological studies^18^. Studies conducted in NHP species have also formally demonstrated resistance to superinfection in these susceptible hosts^9^. Moreover, ZIKV immunity appears effective against both African and Asian lineage strains^19,20^, hence considerable optimism exists for vaccine-induced immunity to protect against ZIKV infection and disease.

Experimental studies using interferon-deficient knockout mice and other strains, have also demonstrated protective efficacy of human neutralising antibodies against infection and subsequent development of microcephaly^21^. Immunodeficient mouse models remain informative despite lacking the ability to fully recapitulate systemic ZIKV infection of immunocompetent hosts, to provide indications of vaccine efficacy^22^. However, due to anatomical differences between NHPs and rodents, including differences in placental structure, comparative pathogenesis studies conducted in multiple NHP species, including New World hosts, suggest higher order mammals may more faithfully reproduce human ZIKV infection and associated immune responses^16,17^.

Whilst there is strong evidence that anti-Zika immune sera may protect, the challenge of measuring the amount of antibody required in a robust comparative manner has prevented clear statements on how strong an anti-ZIKV response is required for vaccine success. Recently a reference material, the 1st WHO International Standard for anti-Asian lineage ZIKV antibody was established to harmonise measurement of these antibodies^23^. Here, we demonstrate that a human anti-Zika convalescent plasma pool calibrated as part of that study passively protected cynomolgus macaques (*Macaca fascicularis*) against challenge with South American-derived Asian lineage ZIKV. Our results provide a framework that supports not only vaccine development but also their early regulatory approval.

## Results

### Standardisation of RT-qPCR assays to detect ZIKV RNA in plasma

To evaluate viral load determinations, data obtained with the RT-qPCR assay were expressed in both ZIKV RNA copies/mL and International Units/ml (IU/mL). Standard curves were generated for both using a plasma RNA series as previously described^16^ and the secondary ZIKV International RNA working reagent (designated NIBSC 16/110) prepared from propagation of the Ugandan 1962 MP1751 strain^24^. This material had been calibrated against the 1st WHO IS for ZIKV RNA as part of an international collaborative study to contain 6.75 IU/mL log_10_^25^.

**Fig. 1:**
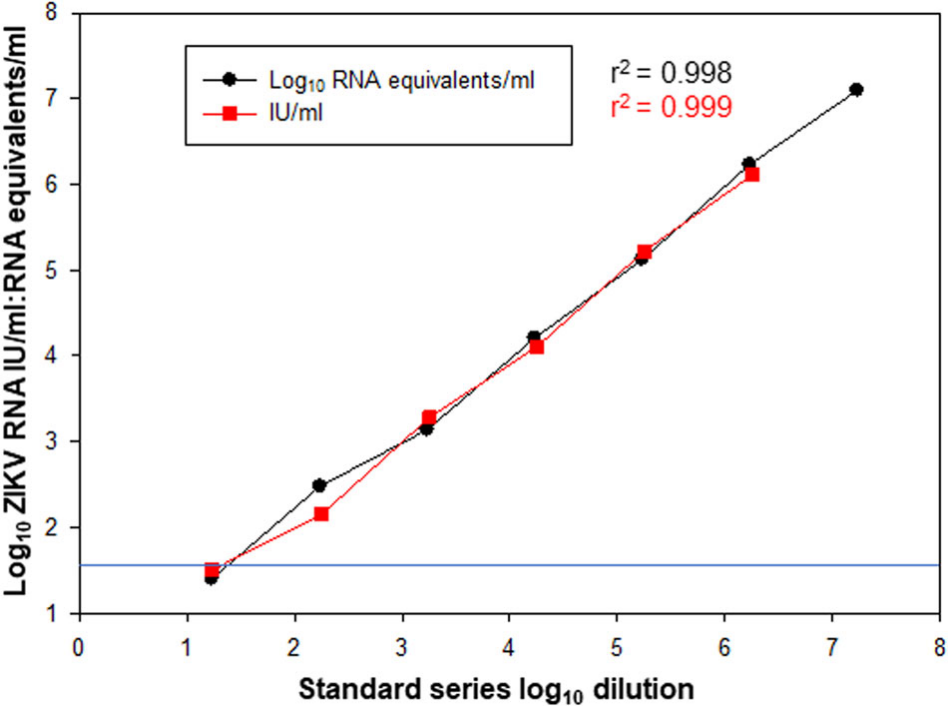
Equivalent amplification efficiency of African and Asian lineage ZIKV by RT-qPCR. Comparison of two ZIKV RNA standard preparations diluted in normal macaque plasma to an extinction end-point. Amplification efficiency of RT-qPCR of ZIKV_PRVABC59_ (black circles) plotted against African lineage (Ugandan 1962 MP1751) QC material (NIBSC 16/110 expressed as IU/ml (red squares). Regression analysis (*r*^2^ values) were 0.998 and 0.999, respectively, with a high degree of agreement between the two reference panels. Limit of detection (LOD) for assays was 50 RNA equivalents/mL as described^16^ and 50 IU/mL.

Initially, ZIKV RNA values were determined using the ZIKV_PRVABC59_ reference virus grown on Vero cells and supernatant virus diluted in negative macaque plasma, generating a standard curve as shown in Fig. 1. In parallel regression analyses, data derived from the external QC material were compared, following serial dilution in macaque-negative plasma. There was a high degree of agreement with parallel regression curves generated for each preparation. Hence, it was possible to convert the ZIKV RNA copies/ml data into IU/mL by interpolation. In this manner, ZIKV RNA could be expressed as RNA equivalents against the WHO measurement system. The limit of detection (LOD) of this assay was determined to be 50 ZIKV RNA copies/mL with the input plasma volume described. Comparable LOD of 50 ZIKV RNA IU/mL were determined by parallel regression analysis which further indicated the RT-qPCR assay to be equally efficient in determining ZIKV RNA values from both African and Asian lineage viruses.

### Immune transfer

Four juvenile cynomolgus macaques were administered human anti-ZIKV polyclonal convalescent plasma via the intra-peritoneal (i.p.) route. The infused material had been prepared from multiple vials of the anti-ZIKV working reagent (NIBSC 16/320). In the international collaborative study that established the 1st International Standard for anti-Asian lineage ZIKV antibodies, the material was determined to have an anti-ZIKV neutralising antibody titre of 2230 NT_50_/mL. Analysis of the study assigned a value of 2756 IU/mL (95% confidence limits of 2003 to 3792 IU/mL) to this preparation^22^. Subsequently, all neutralisation assay data were expressed as a relative potency in IU/mL. Assuming the blood volume/weight ratio of this species is ~66 mL/kg, if 100% antibody was taken up from the peritoneum with no antibody clearance before virus challenge 24 h later, ~76% (±5%) of anti-ZIKV antibody administered i.p. was detected in blood 24 h later. No adverse effects were detected as a result of the immune transfer procedures, with no detectable serum sickness or similar clinical presentations.

### ZIKV RNA is undetectable in blood and tissues of antibody-treated macaques compared with controls

**Fig. 2:**
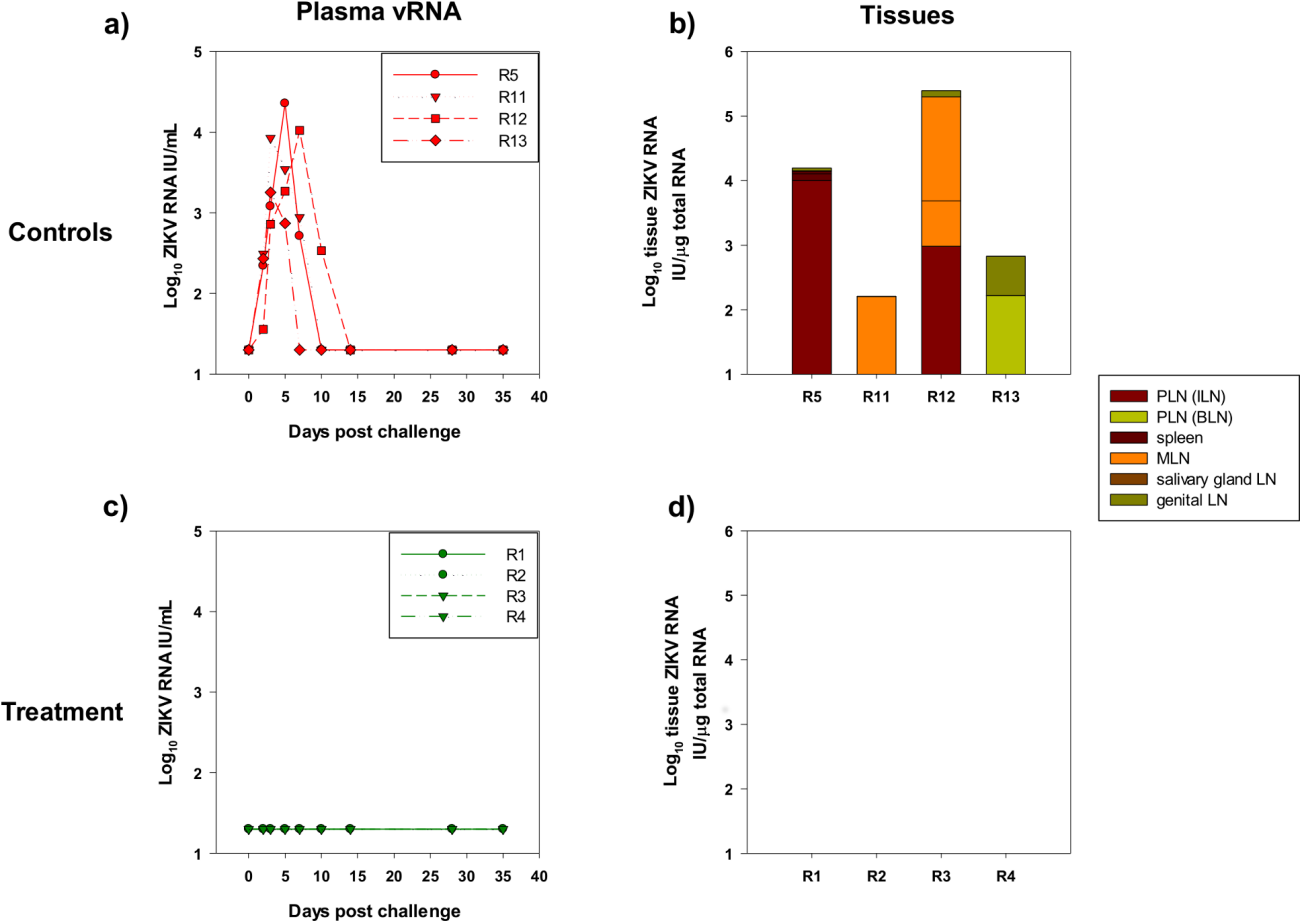
Viral RNA levels in plasma and tissues. **a** Plasma vRNA levels during acute infection during the first 40 days in naïve controls expressed as International Units (IU) /mL. **b** Cell-associated ZIKV RNA levels in tissues, expressed as International Units (IU) per µg total RNA in peripheral lymph nodes (PLN), both inguinal (ILN) and brachial (BLN), spleen, mesenteric (MLN), salivary gland and genital lymph nodes (LN). Macaque R5 was terminated 5 weeks p.i, R11, R12 at 10 weeks p.i. and R13 at 6 weeks p.i. **c** and **d** indicate plasma vRNA and tissue cell-associated RNA levels respectively in immunised macaques (R1–R4).

The primary infection outcome of this study was determined by RT-qPCR analysis of ZIKV RNA levels in plasma. Data for the four untreated, control cynomolgus macaques (R5, R11, R12, R13) are shown in Fig. 2a, with an acute phase viraemia of ~10^4.5^ peaking 3–5 days post inoculation. This is in keeping with previously published findings of ZIKV plasma kinetics in this species^16^. The distribution and levels of ZIKV cell associated-RNA detected at 5 weeks p.i. broadly reflected the acute-phase vRNA kinetics (Fig. 2b). R13 and R11 exhibited the lowest peak vRNA with ZIKV cell-associated RNA sporadically detectable in tissues. R5 and R12, which displayed higher peak plasma vRNA exhibited comparably higher tissue cell-associated RNA loads. The slightly delayed but sustained peak in R12 appeared to be reflected in a proportionately higher level of virus replication in the MLN.

In macaques pre-treated with the convalescent antibody preparation there was no evidence of ZIKV RNA detected either as cell-free viral RNA in plasma or sequestered cell-associated viral RNA in key lymphoid tissues in samples collected at termination (Fig. 2c, d). In blood, there was no evidence of late appearance or delayed viral kinetics (vRNA blips) at subsequent time-points, indicating complete virus suppression at the anticipated time of the acute-phase viraemia. This is further supported by lack of detectable ZIKV genome in any tissue analysed by cell associated-RNA RT-qPCR representing key tissues of the spleen, the iliac, and brachial (peripheral) lymph nodes, or mesenteric, salivary gland and genital lymph nodes. In naïve challenge controls, each of these tissues were genome positive in at least one subject. Lack of detectable virus at any time point in treated macaques underscores the complete suppression of the ZIKV challenge.

### Antibody correlates of protection

**Fig. 3:**
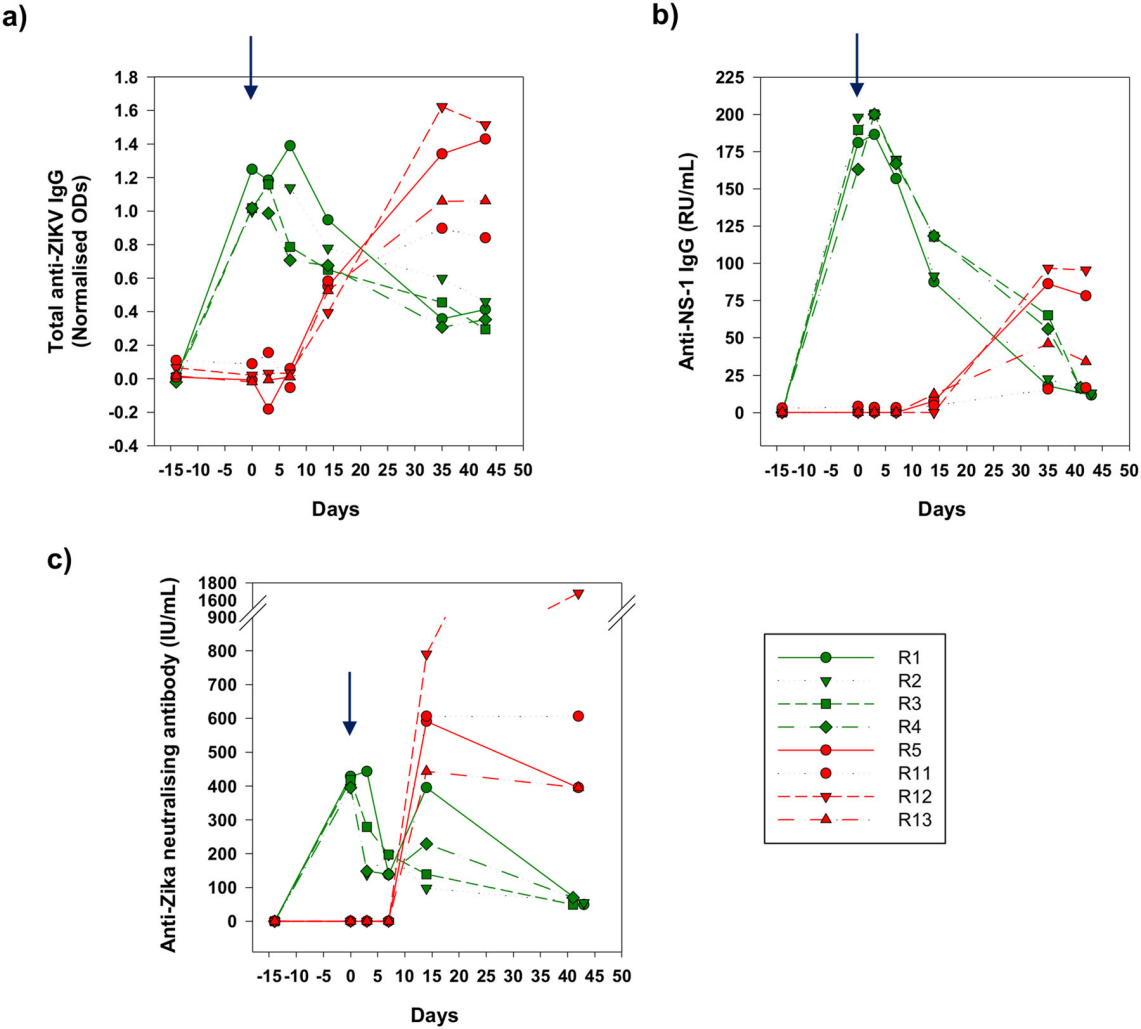
Anti-ZIKV IgG and neutralising antibodies pre and post ZIKV challenge. Sera collected over the time-course were assessed for their anti-ZIKV activity using (**a**) whole virus anti-ZIKV IgG (**b**) binding antibody levels to anti-NS-1 IgG expressed as relative units (RU)/mL, (**c**) neutralisation titres expressed in International Units (IU/mL) Treatment macaques (Group A) are depicted in green and challenge controls (Group B) in red. Arrow indicates administration of the virus.

**Fig. 4:**
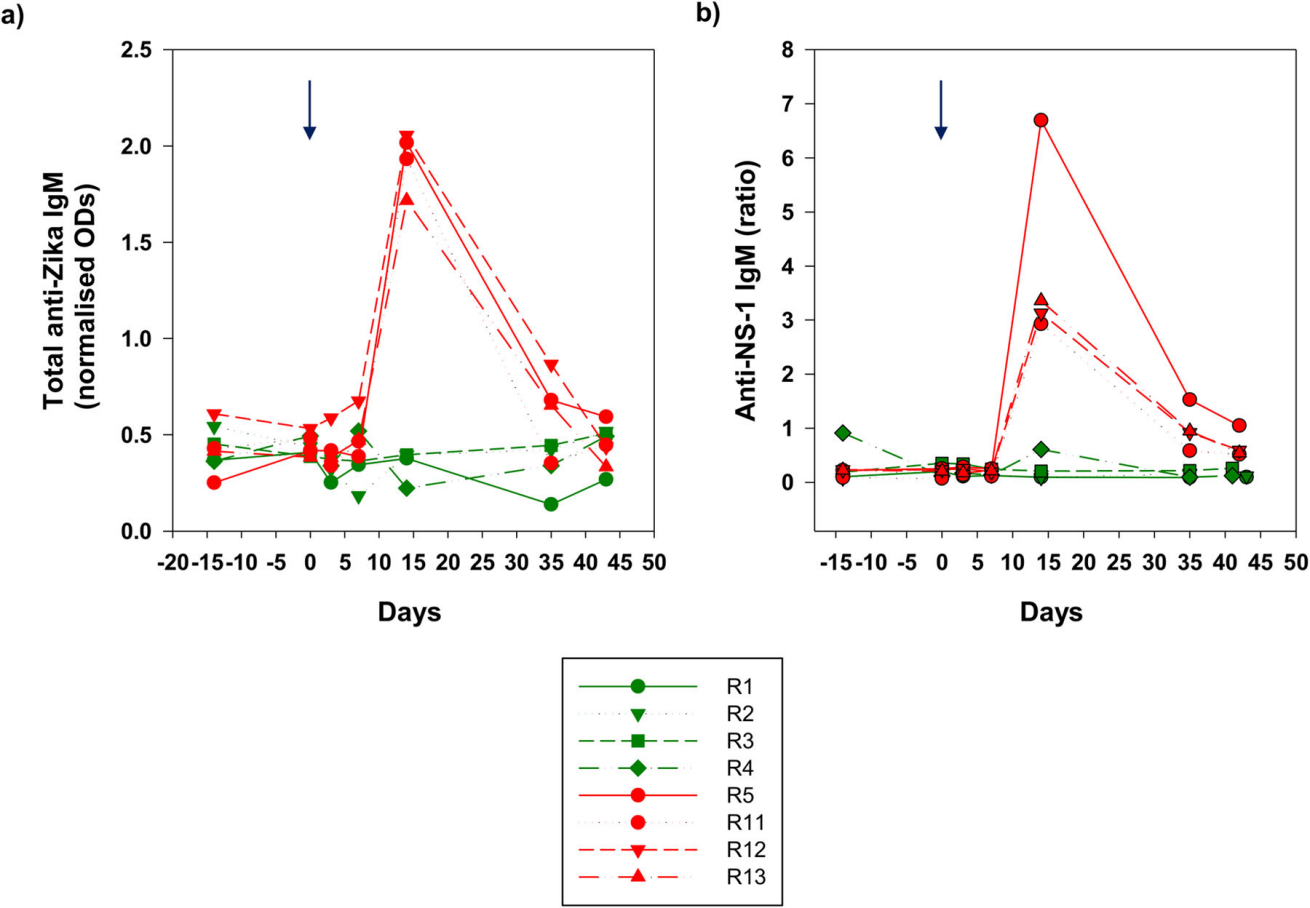
Anti-ZIKV IgM levels. **a** whole viral antigen ELISA and **b** anti-NS-1 binding antibody levels to anti-Zika IgM expressed as a ratio. Arrows indicate time of administration of ZIKV_PRVABC59_ at day 0. Treatment macaques (Group A) are depicted in green and challenge controls (Group B) in red. Arrow indicates administration of the virus.

Antibody responses were compared in both immunised and control macaques using five independent serological assays, summarised in Figs. 3 and 4, measuring IgG and IgM responses to whole virus antigens and the NS-1 protein, as well as neutralising antibody levels. In this manner serological responses were compared pre- and post-administration of ZIKV_PRVABC59_ for the four naïve controls and four macaques receiving passively transferred human plasma.

In the four control macaques (R5, R11–R13), the total levels of anti-Zika IgG and anti-NS-1 specific responses were remarkably similar in the pattern of their dynamic range suggesting the majority of the response detected by the whole virus assay was to NS-1. In these naïve, control macaques challenged with virus, R13 and R5 developed the strongest antibody responses overall post-ZIKV infection. All naïve, challenge controls mounted both a neutralising antibody and an IgM response, the latter detected using the human anti-NS-1 assay and the whole virus macaque-specific IgM assay (Fig. 4). Anti-IgM responses were more consistent using the total IgM assay (Fig. 4a), though there was an exaggerated spike in anti-NS-1-specific IgM in R5 which coincided with highest peak of vRNA in plasma recorded in this individual, further indicating the importance of this protein in the acute response to ZIKV infection.

In contrast, neutralising antibody titres could be compared irrespective of confounding anti-species considerations across the entire study. All controls developed a detectable neutralising antibody response post-ZIKV challenge, which stabilised at 400–600 IU/ml range in 3 macaques, rising to ~1700 IU/ml at 42 days after challenge in macaque R12 (Fig. 3c). In R12 this feature was further reflected in the highest level of total and anti-NS-1 IgG, which correlated with a slightly delayed peak and extended shoulder of plasma vRNA (Fig. 2a). While not apparently inducing a superior IgM response, the antigenic boost sustained during this acute period likely accounts for the very high levels of neutralising and binding antibody levels observed in R12. Taken together, these de novo antibody profiles provided a comparator to outcomes in the immunised macaques receiving passively administered antibody.

In all recipients, 24 h following passive immunisation of the convalescent plasma pool, broadly similar levels of total IgG to whole virus, anti-NS-1 IgG and neutralising antibodies were observed (Fig. 3a–c). The whole virus assay utilising an anti-macaque antibody clearly exhibited significant cross reaction with human anti-Zika IgG. Taken together, this indicates all macaques received very similar levels of transferred immunoglobulin irrespective of the assay used. Hence, on the day of challenge anti-NS-1 IgG levels were between 175–200 relative units (RU)/mL by ELISA and neutralisation levels were ~400 IU/ml. Whole virus antibody titres, expressed as a ratio of normalised ODs, exhibited similarly good reproducibility (macaques R1-R4, all ~1 normalised OD units). Anti-Zika IgM was at or around undetectable levels in immunised macaques at day 0 using both the total anti-Zika IgM and anti-NS-1 assays (Fig. 4**)**. Although a small blip in ZIKV anti-NS-1 IgM was identified in macaque R4 in the pre-immunisation bleed and at 14 days post-challenge, levels remained at or close to the assay cut-off and this phenomenon was not reproduced in the total IgM assay.

Immediately post-ZIKV challenge there was some fluctuation noted in macaque R1 which exhibited a small boost in both neutralising antibody, anti-NS-1, and total IgG levels, detected 3 days post administration of virus (Fig. 3a–c). As a result of this extended peak of antibody, there was a more delayed half-life degradation of antibody levels in R1 which were not subsequently boosted. Similar degradation profiles were evident for R2-R4. Comparable levels of total IgG and anti-NS-1 IgG at 42 days p.i. (and neutralising antibody) suggest an even level of decay across all antibody species. IgG degradation data exhibited a reduction of IgG titres with a half-life of 2–3 weeks (Supplementary Figure 1). Taken together there was no boost in total or anti-NS-1 ZIKV IgG levels noted in response to viral challenge, although in R1 neutralising antibody levels transiently returned to close to their pre-challenge levels. R4 also exhibited a weak increase in neutralising antibodies.

Overall, de novo antibody responses compatible with recent infection with ZIKV_PRVABC59_ were not detected. On administration of virus, the relatively rapid drop in neutralising antibody titres (Fig. 3c) could perhaps be attributed to introduction of the challenge virus. Interestingly, there is a slight deflection back up to ~350 IU/mL in macaque R1 but in this and all macaques there was a subsequent, gradual decay to baseline levels. Anti-IgM responses remained undetectable. Hence, none of the serological responses detected are compatible with an active anti-ZIKV response in any of the immunised macaques.

## Discussion

Establishing immune responses, both in terms of their quality and quantity, that effective vaccines needs to elicit represents valuable data that will accelerate vaccine development and subsequent regulatory approval. Relating outcomes of model systems back to standardised measures of vaccine protection will be important in assessing correlates of vaccine protection elicited by different vaccines. To address this we have utilised human convalescent plasma derived from two ZIKV-exposed individuals during the recent epidemic in central America, who subsequently recovered from infection making a strong humoral immune response. This report describes use of an NHP challenge model of ZIKV infection where prior transfer of a human convalescent plasma pool to recipients receiving 400 IU/mL anti-ZIKV antibodies prevents detectable infection when challenged sub-cutaneously with 1000 pfu ZIKV_PRVABC59_. Passive immunisation of these human antibodies into macaques 24 h prior to sub-cutaneous challenge with the ZIKV_PRVABC59_ strain was able to confer full protection against this contemporary ZIKV strain.

Previous studies have mainly focussed on mouse models which have indicated high titre neutralising antibody responses to envelope antigens are associated with a protective outcome^21^ and passive immunisation with either vaccine-delivered antibodies, virus-specific monoclonal antibodies or convalescent human serum may be efficacious^22^. There is limited data, however, relating to passive protection against ZIKV in immunocompetent NHP systems, although one study conducted in rhesus macaques indicates such an approach may be effective^26^.

Using materials from the international collaborative study used to establish an International Standard in International Units per ml (IU/ml), we were able to determine that a neutralising antibody titre of 400 IU/ml was sufficient to deliver complete protection. The protection demonstrated appeared complete, with no evidence of ZIKV RNA in the blood or tissues, or detection of antibody responses apart from those directly attributable to the passive injection, prior to challenge with wild-type virus. As no cellular component was transferred, this reinforces the notion that antibodies present in the cell-free component of blood alone were responsible for the protection. These data are very valuable for both vaccine developers and regulators which provide a mechanism of protection candidate vaccines need to replicate. In addition, they provide some guidance on the amount of antibody required at the time of virus challenge to confer complete protection against detectable infection. These data may assist in establishing the likely dosing schedule to elicit robust protection and in addition the likely duration of protection conferred by candidate vaccines. At the same time since a titration of the reference serum was not performed, we do not know the lower limit of antibody required to confer complete protection. The size, blood volume, cost, and welfare issues of using Old World non-human primate models of Zika virus infection may mean that alternative models should be used to undertake these plasma/serum titration studies. Smaller New World NHP species demonstrated to be susceptible to ZIKV may be suitable for this purpose.

Both immunocompetent and immunodeficient mouse strains support replication of ZIKV in vivo. It should also be noted that the current study was conducted in cynomolgus, rather than rhesus macaques. The peak viremia and tissue distribution of ZIKV in the former species is less than the latter^16^. Thus, these studies may overestimate the degree of protection possible with plasma/serum alone in more susceptible NHP species. Nevertheless, the key anatomical similarities between NHPs and humans, for example in placental structure which indicate that some further studies of protection should be performed in an appropriate NHP species, particularly those evaluating protection against congenital Zika syndrome. The role antibody transfusion plays during an active, acute infection or as a pre-exposure prophylaxis therapy could also be explored and would provide further valuable information, although the timing of such intervention would be critical given the rapid establishment of the virus immediately post-exposure and the natural decline of antibody titres post immunisation.

Here, we have formally demonstrated complete protection from ZIKV challenge in a model system using human polyclonal convalescent immune serum. Complete protection is presumed to have occurred since there were no de novo antibody responses to ZIKV in any of the assays used and all macaques remained ZIKV RNA genome negative. The whole virus anti-ZIKV IgG and IgM assays which employed anti-macaque secondary antibodies failed to detect any evidence for an acute or systemic ZIKV infection. Rapid loss of neutralising titres on challenge were compatible with a significant reduction in the infective potential of the inoculum, either removing the ability of the virus to infect completely or reducing levels to below a sub-clinical threshold. Our previous studies characterising the early kinetics of ZIKV infection in different NHP species, including cynomolgus macaques, demonstrate rapid uptake and widespread dissemination of virus 3 days p.i.^16^. Although it cannot be completely excluded that very low levels of persisting sequestered virus may exist at some sites, we found no evidence of viral RNA in either the blood or the major lymphoid tissues sampled which would indicate this has occurred. Coupled with lack of any sustained antigenic re-exposure the serological data would appear to concur with this observation.

In this study, both antibody and viral challenge outcome data were expressed with reference to International Units (IUs), paving the way for standardisation of vaccine response data across different studies and vaccine regimens. This important consideration in determining relative vaccine efficacy and potency of different vaccine approaches and likely ability to protect in vivo, may ultimately obviate the need for extensive pre-clinical challenge data to be generated for candidate vaccines. Use of an immunocompetent host where both immune response and virus dissemination properties in unvaccinated, naive individuals resembles that of ZIKV infection in humans provides a distinct advantage over previously published studies in rodents. However, different vaccine platforms may require a more tailored approach although this does provide a framework for comparative studies.

On the face of it, vaccines that generate a strong neutralising antibody response to ZIKV represent a clear goal for ZIKV immunisation studies via active vaccination. Responses to sub-unit components of the virus representing the external domain of the ZIKV envelope (ZEDIII) may provide effective cross-neutralising activity^27,28^. However, some caution may be needed in this area given that cases of ZIKV-microcephaly have been strongly correlated to ZIKV neutralising antibody responses in previously ZIKV-naïve individuals. Prior cross-reactive antibody reactivity to other flaviviruses do not appear to be linked to an antibody-dependent enhancement (ADE)-related pathologic mechanism but may be intrinsic to Zika ADE generation in naïve, susceptible hosts. Maternal antibodies with enhanced reactivity to ZEDIII antigens and ZIKV neutralisation per se have been strongly associated with cases of microcephaly in the recent outbreak, accounting for the most severe outcomes of ZIKV positive pregnancies^29^. The caveat for any intervention strategy must be that if only partial protection was attained by vaccination where otherwise naïve subjects still became infected where a ZIKV neutralising antibody response was generated but failed to deliver complete protection from infection, potentiation of subsequent infections where boosts in anti-ZIKV titres that may be deleterious could result in adverse outcomes. Low level or non-neutralisation titres in the context of homotypic ZIKV ADE potentiating ZIKV pathogenesis is thus both a complex and important area^30^, which may need factoring into the regulatory understanding of both beneficial and deleterious responses. Further studies of ZIKV immunisation should address this issue encompassing the broadest range of anti-ZIKV antibody responses, both in terms of quality as well as quantity, to enable differentiation between protective and potentially harmful anti-ZIKV responses that could be elicited by vaccination. In future studies, inclusion of other polyclonal immunoglobulin preparations, possibly including those from commercial sources, would provide an opportunity to assess the potential role of non-ZIKV specific antibodies with the potential to block viral epitopes.

The data presented here establish the potential for a human convalescent plasma reference material to calibrate the threshold levels of anti-ZIKV antibodies needed for vaccine protection in an NHP model. These data may enable effective standardisation of vaccine potency that will permit meaningful comparison between different vaccine strategies designed to generate protective immunity, identified as a key part of vaccine development^31^. This will lead to harmonised evaluation of candidate vaccines and the accelerated regulatory approval of safe, efficacious vaccines against Zika virus. Combined with sera/plasma from human clinical trials clarification of protective efficacy would certainly facilitate candidate vaccine evaluation. This approach may prove to be particularly useful in the screening of the raft of vaccines currently being developed to the SARS-CoV-2 coronavirus and perhaps other emerging viruses, to a provide scientific basis and rationale for the selection of the most promising vaccines.

## Methods

### Ethical statement

All animal procedures were performed in strict accordance with UK Home Office guidelines, under licence 70/8953 granted by the Secretary of State for the Home Office which approved the work described, in accordance with local ethical guidelines and internal committee approval for animal welfare at NIBSC. This study conforms to all relevant ethical regulations for animal work in the UK. Purpose-bred, juvenile cynomolgus macaques (*Macaca fascicularis*) were group housed for the duration of the study, with daily feeding and access to water ad libitum. Regular modifications to the housing area and environmental enrichment of all study NHPs were made by husbandry staff. Environmental temperature was appropriate for macaque species and rooms subject to 12 hr day/night cycles of lighting. Animals were acclimatised to their environment and deemed healthy by the named veterinary surgeon prior to inclusion on the study. All surgical procedures were performed under anaesthesia with recovery.

### Study design

Eight juvenile Mauritian-origin cynomolgus macaques were used according to ethical guidelines. Antibody sourced for the study was a pool of polyclonal convalescent plasma obtained from two donors originating in the Dominican Republic, supplied by Boca Biolistics Ltd, Florida, USA. This material had been pre-prepared as an anti-Zika working reagent (NIBSC 16/320) whereby multiple lyophilised vials were reconstituted to derive a pool of convalescent plasma, characterised as part of a collaborative study of anti-Zika antibodies^23^. Results from this collaborative study assigned a value of 2756 IU/mL (95% confidence limits of 2003 to 3792 IU/mL). A total of 25 mL of reconstituted plasma was administered intra-peritoneally (i.p.) into each of four cynomolgus macaques weighing 2.2–2.4 kg. Intra-peritoneal injection was used to obviate the need to concentrate the Ig fraction and administer serum/plasma at volumes achieving circulating anti-Zika antibody concentrations 1:2 to 1:4 of that in the administered plasma with an uptake within 24 h post administration. All four, and an additional four naïve challenge controls were subsequently administered with 10^3^ Pfu/mL of the PRVABC59 Zika isolate via the subcutaneous route as previously described^16^, representing a biologically relevant challenge^32^. Calculations were according to accepted criteria, assuming a circulating volume of 66 mL/kg for cynomolgus macaques^33^.

### Quantitative RT-qPCR for ZIKV RNA in plasma

Quantitative RT-qPCR was performed on total RNA extracted from 140ul plasma as previously described^16^. The limit of detection for the RT-qPCR was 50 RNA IU/mL of plasma, determined by end-point analysis of target molecules. All data were visualised using the SigmaPlot v12 statistical package.

### Cell-associated ZIKV levels in tissues

Total RNA (100 ng) isolated from selected tissues samples of peripheral lymph nodes ILN, BLN), spleen, MLN, salivary gland LN, and genital LN were subjected to RT-qPCR, normalised to GAPDH, as previously described^16^. Data were expressed as IU/µg total RNA with a limit of detection of 20 IU/µg total RNA.

### Anti-Zika serology

Anti-Zika neutralising antibodies were determined as previously described^16^, with values expressed as International Units per mL (IU/mL) serum. Anti-NS-1 IgG and IgM levels were determined using commercially available assays (EuroImmune Ltd), performed according to the manufacturer’s instructions. Total anti-Zika responses to whole virus antigens were also assessed. In brief, EIA plates (Nunc, Maxisorb) were coated overnight at 4 °C with whole virus ZIKV_PRVABC59_ supernatant diluted in phosphate buffered saline (PBS) representing a concentration of 1 × 10^5^ pfu/mL, fixed with 4% paraformaldehyde for 30 min at RT °C. Supernatant from uninfected Vero E6 cells represented control antigens. Plates were washed, blocked with 10% FCS in PBS for 1 h at RT °C, washed with PBS/0.05% Tween-20. Samples were diluted 1:50 in 10% FCS/PBS blocking buffer, added to the plate, incubated for 1 h at RT^o^C and washed (x3) in PBS-0.05% Tween-20. Secondary antibody detection was with anti-macaque IgG or anti-macaque IgM-(μ-chain specific) conjugated to horseradish peroxidase (Sigma Ltd), diluted 1:2000 or 1:5000 respectively in 5% FCS in PBS and incubated for 1 h at RT °C. Unbound secondary antibody was removed by washing (x3) with PBS-0.05% Tween-20 and visualised by addition of TMB for 5 mins; reactions were stopped by addition of 2 M H_2_SO_4_. Absorbance was read at 450 nM. Normalised optical density (OD) readings were derived by subtracting control from test antigens.

### Reporting summary

Further information on research design is available in the Nature Research Reporting Summary linked to this article.

## Supplementary information

Supplentary Figure 1

Reporting Summary

## Data Availability

All data generated or analysed during this study are included in this published article (and its supplementary information files).
