# Supplementary material for: Passive immunisation of convalescent human anti-Zika plasma protects against challenge with New World Zika virus in cynomolgus macaques
Source: NPJ Vaccines. 2020 Sep 15;5:86. doi: 10.1038/s41541-020-00234-y (PMC7492244; doi:10.1038/s41541-020-00234-y)
Supplement: Supplementary file 1 — Supplentary Figure 1 [file 41541_2020_234_MOESM1_ESM.pdf]

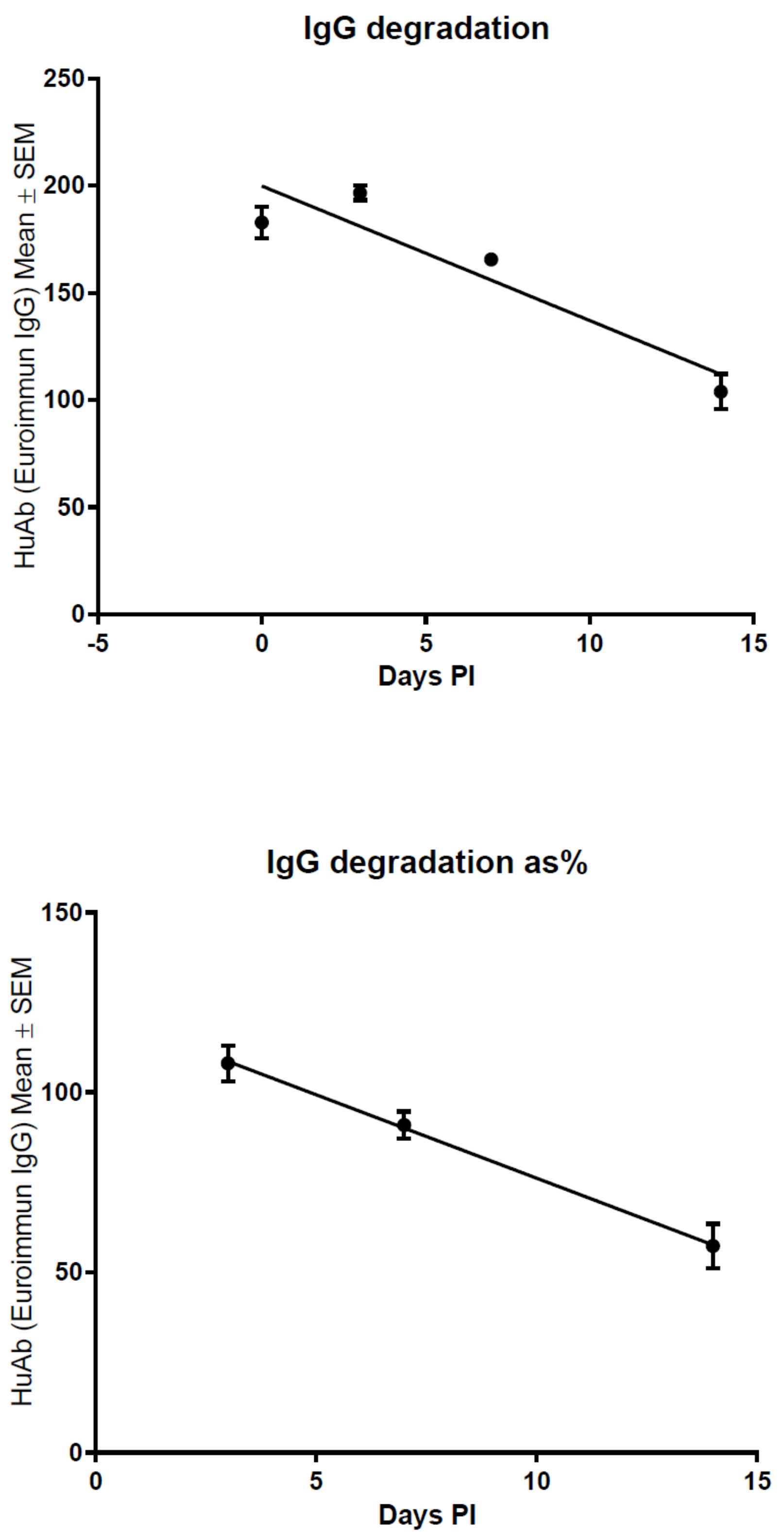

Supplementary Figure 1.

IgG degradation curve for human antibody (HuAb) IgG days post-infection expressed as relative units plotted against days post-infection (PI). Standard error of the mean (SEM) for replicate values are shown.
