# Supplementary material for: Passive immunisation of convalescent human anti-Zika plasma protects against challenge with New World Zika virus in cynomolgus macaques
Source: NPJ Vaccines. 2020 Sep 15;5:86. doi: 10.1038/s41541-020-00234-y (PMC7492244; doi:10.1038/s41541-020-00234-y)
Supplement: Supplementary file 2 — Reporting Summary [file 41541_2020_234_MOESM2_ESM.pdf]

nature research

Corresponding author(s): Neil J Berry
Last updated by author(s): 8/8/2020

Reporting Summary

Nature Research wishes to improve the reproducibility of the work that we publish. This form provides structure for consistency and transparency in reporting. For further information on Nature Research policies, see our Editorial Policies and the Editorial Policy Checklist.

Statistics

For all statistical analyses, confirm that the following items are present in the figure legend, table legend, main text, or Methods section.

n/a	Confirmed	
☐	☒	The exact sample size (n) for each experimental group/condition, given as a discrete number and unit of measurement
☐	☒	A statement on whether measurements were taken from distinct samples or whether the same sample was measured repeatedly
☒	☐	The statistical test(s) used AND whether they are one- or two-sided<br>*Only common tests should be described solely by name; describe more complex techniques in the Methods section.*
☒	☐	A description of all covariates tested
☒	☐	A description of any assumptions or corrections, such as tests of normality and adjustment for multiple comparisons
☒	☐	A full description of the statistical parameters including central tendency (e.g. means) or other basic estimates (e.g. regression coefficient) AND variation (e.g. standard deviation) or associated estimates of uncertainty (e.g. confidence intervals)
☒	☐	For null hypothesis testing, the test statistic (e.g. F, t, r) with confidence intervals, effect sizes, degrees of freedom and P value noted<br>*Give P values as exact values whenever suitable.*
☒	☐	For Bayesian analysis, information on the choice of priors and Markov chain Monte Carlo settings
☒	☐	For hierarchical and complex designs, identification of the appropriate level for tests and full reporting of outcomes
☒	☐	Estimates of effect sizes (e.g. Cohen's d, Pearson's r), indicating how they were calculated

Our web collection on statistics for biologists contains articles on many of the points above.

Software and code

Policy information about availability of computer code

Data collection: No software was used

Data analysis: No software was used

For manuscripts utilizing custom algorithms or software that are central to the research but not yet described in published literature, software must be made available to editors and reviewers. We strongly encourage code deposition in a community repository (e.g. GitHub). See the Nature Research guidelines for submitting code & software for further information.

Data

Policy information about availability of data

All manuscripts must include a data availability statement. This statement should provide the following information, where applicable:

- Accession codes, unique identifiers, or web links for publicly available datasets
- A list of figures that have associated raw data
- A description of any restrictions on data availability

All data generated or analysed during this study are included in this published article (and its supplementary information files).

Field-specific reporting

Please select the one below that is the best fit for your research. If you are not sure, read the appropriate sections before making your selection.

☒ Life sciences ☐ Behavioural & social sciences ☐ Ecological, evolutionary & environmental sciences

For a reference copy of the document with all sections, see nature.com/documents/nr-reporting-summary-flat.pdf

Life sciences study design

All studies must disclose on these points even when the disclosure is negative.

Sample size	Sample sizes were based on historical studies for this type of study, as an accepted minimum criteria within the field.
Data exclusions	No data were excluded from the analyses
Replication	Due to limits with working with NHPs, the study was performed once and reported herein. Validated laboratory assays were used to verify the study outcomes.
Randomization	This is not relevant to the study comprising two groups of four non-human primates.
Blinding	This is not relevant to the study comprising two groups of four non-human primates.

Reporting for specific materials, systems and methods

We require information from authors about some types of materials, experimental systems and methods used in many studies. Here, indicate whether each material, system or method listed is relevant to your study. If you are not sure if a list item applies to your research, read the appropriate section before selecting a response.

Materials & experimental systems

n/a	Involved in the study	
☐	☒	Antibodies
☐	☒	Eukaryotic cell lines
☒	☐	Palaeontology and archaeology
☐	☒	Animals and other organisms
☒	☐	Human research participants
☒	☐	Clinical data
☒	☐	Dual use research of concern

Methods

n/a	Involved in the study	
☒	☐	ChIP-seq
☒	☐	Flow cytometry
☒	☐	MRI-based neuroimaging

Antibodies

Antibodies used	Anti-monkey IgG peroxidase antibody, rabbit (Sigma-Aldrich), Cat No: A2054, polyclonal; anti-monkey IgM, goat antibody, (Sigma-Aldrich), Cat no: SAB3700780; polyclonal, lot number R-139502
Validation	Validation on manufacturer's website and in house validation

Eukaryotic cell lines

Policy information about cell lines

Cell line source(s)	Vero E6
Authentication	The cell line was available in house from our repository
Mycoplasma contamination	All cell lines were negative for mycoplasma
Commonly misidentified lines (See ICLAC register)	none applicable

Animals and other organisms

Policy information about studies involving animals; ARRIVE guidelines recommended for reporting animal research

Laboratory animals	Cynomolgus macaque (Macaca fascicularis) , Mauritian-origin; 5 males, 3 females; juveniles
Wild animals	The study did not involve wild animals
Field-collected samples	The study did not involve samples collected from the field.
Ethics oversight	The study was performed in accordance with UK Home Office regulations and subjected to local ethical committe reviewr and approval (NIBSC AWERB ctte; animal welfare ethical review board).

Note that full information on the approval of the study protocol must also be provided in the manuscript.
